# Dietary choline and betaine intake, cardio-metabolic risk factors and prevalence of metabolic syndrome among overweight and obese adults

**DOI:** 10.1186/s12902-023-01323-4

**Published:** 2023-03-27

**Authors:** Mohammad Sadegh Pour Abbasi, Ayda Zahiri Tousi, Yalda Yazdani, Sahar Vahdat, Farshad Gharebakhshi, Negin Nikrad, Ali Manzouri, Abnoos Mokhtari Ardekani, Faria Jafarzadeh

**Affiliations:** 1 Department of Cardiovascular Surgery, Kashan University of Medical Sciences, Kashan, Iran; 2 Razavi Cancer Research Center, Razavi Hospital, Imam Reza International University, Mashhad, Iran; 3 Immunology Research Center, Tabriz University of Medical Sciences, Tabriz, Iran; 4 Isfahan Kidney Disease Research Center, School of Medicine, Khorshid Hospital, Isfahan University of Medical Sciences, Isfahan, Iran; 5 Department of Radiology, School of Medicne, Imam Hossein Hospital, Shahid Beheshti University of Medical Sciences, Tehran, Iran; 6 Student Research Committee, Tabriz University of Medical Sciences, Tabriz, Iran; 7 Health Policy Research Center, Shiraz University of Medical Sciences, Shiraz, Iran; 8 Endocrinology and Metabolism Research Center, Institute of Basic and Clinical Physiology Science, & Physiology Research Center, Kerman University of Medical Sciences, Kerman, Iran; 9 Department of Internal Medicine, School of Medicine, North Khorasan University of Medical Sciences, Bojnourd, Iran

**Keywords:** Choline, Betaine, Blood pressure, Lipid profile, Metabolic syndrome

## Abstract

**Background:**

Choline is an important metabolite involved in phospholipids synthesis, including serum lipids, and is the immediate precursor of betaine. There are numerous studies with inconsistent results that evaluated the association between dietary choline intakes with cardiovascular risk factors. In addition, the association between dietary betaine and choline intakes with cardio-metabolic risk factors is not well studied. In the current study, our aim was to evaluate dietary choline and betaine intakes in the usual diet of obese individuals and to assess its association with serum lipids, blood pressure and glycemic markers among obese individuals.

**Methods:**

We recruited a total number of 359 obese people aged between 20 and 50 years in the present study. A semi-quantitative food frequency questionnaire (FFQ) was used for dietary assessment; dietary choline and betaine intakes were calculated using the United States Department of Agriculture (USDA) database. National cholesterol education program adult treatment panel (NCEP-ATP)-III criteria was used metabolic syndrome (MetS) definition. Enzymatic methods were used to assess biochemical variables. Body composition was measured with the bioelectrical impedance analysis (BIA) method.

**Results:**

Higher body mass index (BMI), waist to hip ratio (WHR), fat-free mass (FFM) and basal metabolic rate (BMR) were observed in higher tertiles of dietary choline intake (P < 0.01). There was no significant difference in terms of biochemical parameters among different tertiles of dietary choline intake, while systolic blood pressure (SBP) and diastolic blood pressure (DBP) were reduced in higher betaine tertiles (P < 0.05). For total dietary choline and betaine intakes, there was a reduction in DBP and low density lipoprotein (LDL) concentrations (P < 0.05). Also, a non-significant reduction in serum total cholesterol (TC), triglyceride (TG) and MetS prevalence was observed in higher tertiles of dietary choline and betaine intakes. After classification of the study population according to MetS status, there was no significant difference in biochemical variables in subjects with MetS (P > 0.05), while in the non-MetS group, SBP, DBP, TG and insulin levels reduced in higher tertiles of dietary betaine and choline (P > 0.05).

**Conclusion:**

According to our findings, higher dietary intakes of choline and betaine were associated with lower levels of blood pressure and LDL concentrations among obese individuals. Further studies are warranted to confirm the results of the current study.

## Introduction

Obesity is considered as one of the most important health problems worldwide and its prevalence is growing in different geographical regions [1]. The worldwide number of overweight and obese adults in 2014, was more than 1.9 billion and 600 million adults respectively [2]. Alongside of increased obesity prevalence, the occurrence of non-communicable disease (NCDs) is also increasing mostly because of changes in lifestyle and dietary behaviors [3]. In Iran, increased obesity prevalence mostly is attributed to nutrition transition and the combined prevalence of overweight and obesity may be as high as 76% in some regions [4–6]. Diet, is a modifiable risk factor for chronic disease and in recent years, numerous studies have been published focusing on the role of healthy adequate diet in diet-disease relationships [7–10]. Numerous studies have focused on the relationship between single dietary ingredients (e.g. isolate effects of vitamins or minerals) [11–14], or the role of dietary patterns [15–19] dietary indices (e.g. glycemic indices, inflammatory indices, etc.) [17, 20–22] or herbal medicine [23–25] in developing obesity and related disorders; but, very limited number of studies have evaluated the role of dietary compounds like betaine and choline in obesity-related comorbidities.

Choline and betaine are quaternary ammonium compounds that are synthesized from diet or de novo synthesis in tissues; although an insufficient diet can develop choline deficiency [26, 27]. Choline is considered as the primary source of methyl groups in the diet, and its major dietary sources are eggs, sea foods, milk, liver and beef [28], while betaine is mostly, obtained from cereals and grains, beets and spinach, shrimp, wheat germ, wheat bread, and raw mushrooms [29–31]. Choline has numerous roles in the body such as its role in membrane phospholipids, like phosphatidylcholine, choline plasmalogens, and sphingomyelin, acting as cholinergic neurotransmission, platelet-activating-factor formation, hepatic secretion of very low density lipoprotein cholesterol (VLDL), and methyl transport [32]. Choline is a potent methyl donor that produces betaine through oxidation and betaine functions as a compatible osmolyte and methyl donor in many pathways, including the homocysteine methylation [33]. Numerous studies, have investigated the beneficial effects of dietary betaine and choline on body composition or cardio-metabolic markers; the results of the studies evaluating the effects of dietary choline and betaine on anthropometric measurements like body mass index (BMI) or fat mass (FM) are inconsistent [34–37]. The results of the studies evaluating the effects of dietary choline or betaine intake on cardiovascular risk factors (e.g. blood pressure or lipid profile) are more consistent; while several studies showed that an increase in dietary choline intake was associated with a reduced prevalence of hypercholesterolemia [35] and reduced risk of ischemic stroke [38], some others showed no association between dietary betaine and choline intakes with cardiovascular disease (CVD) risk factors [39] and its incidence [40]. Other studies reported more favorable glycemic markers and lipid profile in higher dietary intakes of choline and betaine; in the study by Gao X et al. [41], higher dietary choline and betaine intakes were associated with lower insulin resistance. In a population-based study among 2332 male participants that was performed by Virtanen JK et al., [42] dietary choline and phosphatidylcholine intakes were associated with reduced diabetes risk; while in two other population-based studies, higher dietary choline and betaine intakes were associated with increased diabetes risk [37, 43]. Therefore, there is a great between-study heterogeneity regarding the association between dietary choline and betaine intakes and metabolic parameters in different studies that is possibly due to differences in the disease status or geographical distributions; moreover, no study is available to evaluate this hypothesis in obese individuals in Tabriz and Tehran cities of Iran. Obesity is the origin of numerous comorbidities and obese individuals are at greater risk of numerous diseases. Therefore, in the current study, we aimed to investigate the association between dietary choline and betaine intakes with metabolic parameters including lipid profile, glycemic markers, blood pressure and risk of metabolic syndrome among obese adults in Iran.

## Methods and materials

### Participants

A cross-sectional study was conducted among 359 obese individuals in Tabriz and Tehran cities, Iran. Study subjects were invited by public announcements and were included if they met inclusion criteria (e.g. being aged 20 to 50 years old, BMI ≥ 30 kg/m^2^). The exclusion criteria were: being pregnant, lactating, menopause, having recent bariatric surgery, or CVD, cancer, hepatic and renal diseases, diabetes mellitus, and taking any weight-affecting medications. Full-informed approved written consent was taken from all of the participants and the study proposal was approved by the Ethics Committee of Tabriz University of Medical Sciences (Code: IR.TBZMED.REC.1401.648).

### General characteristics and anthropometric assessments

Socio-demographic information including sex, age, smoking status, education attainment, marital status, occupation, medical histories, and family size were obtained via questionnaire; then, socioeconomic status (SES) score was calculated by quantifying the scores of occupation, educational status, family size and home ownership as individual indicators that were ranked from lowest to highest. Body composition measurements was done by bioelectrical impedance analysis (BIA) method (Tanita, BC-418 MA, Tokyo, Japan). Participant’s height and weight were measured using a wall-mounted stadiometer and a Seca scale (Seca co., Hamburg, Germany) to the nearest 0.5 cm and 0.1 kg respectively. Short form of the International Physical Activity Questionnaire (IPAQ) was used for physical activity assessment [44–46]. Waist circumference (WC) was measured at the midpoint between the lower costal margin and the iliac crest using a tape measure to the nearest 0.1 cm while hip circumference (HC) was measured over the widest part of the buttocks and was recorded to the nearest 0.1 cm. BMI and waist-to-hip ratio (WHR) were calculated. Blood pressure was measured with a standard mercury sphygmo-manometer twice in the same arm after at least 15 min of rest and then mean of the two measurements was used for analysis. Metabolic syndrome (MetS) was defined according to the national cholesterol education adult treatment panel (NCEP-ATP) - III criteria [47–49].

### Dietary assessments

Dietary information was collected using a validated semi-quantitative food frequency questionnaire (FFQ), adapted for Iranian population [50]. Participants were asked to report the frequency and amount of each food item consumed on a daily, weekly, monthly or yearly basis. Then, the reported frequency of consumed foods and portion sizes for each food item were converted to gram using household measures. Choline, glycero-phospho-choline, phospho-choline, phosphatidyl-choline, and betaine were calculated by multiplying each food item based on the United States Department of Agriculture (USDA) food content databases [51]. Total choline intake was calculated as the sum of choline intake from free choline, glycero-phospho-choline, phospho-choline, and phosphatidyl-choline. The sum of total choline and betaine together was used to calculate total choline and betaine intake.

### Biochemical assessment

A 10 ml venous blood samples was obtained from each subject and centrifuged at 4500 rpm for 10 min to separate serum and plasma. Serum total cholesterol (TC), triglyceride (TG), high-density lipoprotein cholesterol (HDL-C), and fasting blood sugar (FBS) were evaluated using commercial kits (Pars Azmoon, Tehran, Iran). Furthermore, low-density lipoprotein cholesterol (LDL-C) level was estimated by the Friedewald equation [52]. Enzyme-linked immunosorbent assay kits were used to measure serum insulin, concentrations (Bioassay Technology Laboratory, Shanghai Korean Biotech, Shanghai City, China). Homeostatic model assessment for insulin resistance (HOMA-IR) was calculated using the formula: fasting insulin (µ IU/ml) × fasting glucose (mmol/l) /22.5 and quantitative insulin sensitivity check index (QUICKI) as 1/ [log fasting insulin (µU/mL) + log glucose (mmol/L)].

### Statistical analyses

Statistical Package for Social Sciences (version 21.0; SPSS Inc, Chicago IL) was used for data analysis. Data were represented as mean ± SD and frequency and percent for continuous and discrete quantitative variables. The comparison of continuous and discrete quantitative variables across tertiles of dietary choline, betaine and total choline and betaine intakes were performed using Chi-square and one-way analysis of variance (ANOVA) respectively. Analysis of co-variance (ANCOVA) was used for comparison of biochemical variables after adjustment for confounders (age, sex, BMI, PA, history of CVD, smoking and total energy intake).

## Results

The comparison of general characteristics and anthropometric features among different tertiles of dietary choline, betaine and total choline and betaine intakes are presented in Table 1. There was a total of 57.9% males and 41.5% females in the current study. As shown, BMI, WHR, fat free mass (FFM) and BMR were higher in higher tertiles of dietary choline intake (P < 0.01). WHR was also higher in higher tertiles of dietary betaine intake than in lower tertile. For total dietary choline and betaine intakes, BMI, WC and FFM were higher in highest tertiles. The comparison of dietary energy and nutrient intakes across tertiles of dietary betaine and choline intakes is presented in Table 2. There was an increase in almost all of the dietary micronutrients’ intake in higher tertiles of dietary choline, betaine and total choline and betaine intakes (P < 0.001). In Table 3, the comparison of serum lipids and glycemic markers across different tertiles of dietary choline, betaine and total choline and betaine intakes is shown in Table 3. As shown, no significant difference in terms of biochemical parameters in different tertiles of dietary choline intake was observed, while there was a reduction in SBP and DBP in higher betaine tertiles (P < 0.05). For total dietary choline and betaine intakes, there was a decrease in DBP and LDL concentrations (P < 0.05). A clinically significant but statistically non-significant reduction in serum TC and TG was also observed by increased dietary choline, betaine and total choline and betaine intakes. As shown in Table 4, after classification of study population into two groups based on MetS status, no significant difference was observed in any of the biochemical variables in individuals with MetS by tertiles of dietary choline, betaine and total choline and betaine intakes (P.0.05), while in individuals without MetS, in higher tertiles of dietary choline, betaine and total choline and betaine intake, lower levels of SBP and TG were observed. In higher tertiles of dietary betaine and total choline and betaine intakes, lower levels of DBP was observed. Also, in non-MetS individuals, increased total choline and betaine intakes were accompanied with reduced serum insulin concentrations. Results of the biochemical variables were achieved after adjustment for age, BMI, physical activity level, smoking, history of CVD and total energy intake.

**Table 1:** General characteristics and anthropometric measurements of study participants across different tertiles of dietary choline, betaine and total choline and betaine intake

Variables		Total choline				Total betaine				Total choline and betaine			
		1st tertile (n = 112)	2nd tertile (n = 113)	3rd tertile (n = 113)	P	1st tertile (n = 112)	2nd tertile (n = 113)	3rd tertile (n = 113)	P	1st tertile (n = 112)	2nd tertile (n = 113)	3rd tertile (n = 113)	P
**Age (year)**		41.20 (9.54)	40.41 (8.46)	39.96 (9.16)	0.583	41.67 (9.24)	40.53 (9.34)	39.08 (8.39)	0.056	41.94 (9.50)	40.57 (8.68)	39.07 (8.81)	0.059
**Weight (kg)**		89.26 (14.23)	92.07(14.86)	94.86 (14.03)	**0.015**	89.92 (16.80)	92.20 (13.77)	94.08 (12.49)	0.099	88.97 (15.60)	91.38 (13.89)	95.83 (13.25)	**0.001**
**Height (cm)**		165.90 (9.85)	168.36 (9.78)	169.44 (9.64)	**0.022**	167.53(10.36)	168.05 (9.56)	167.91 (9.67)	0.881	166.93 (10.26)	167.47 (9.23)	169.32 (9.94)	0.161
**BMI (kg/m** ^**2**^ **)**		32.42 (4.97)	32.50 (4.75)	33.07 (4.85)	**0.050**	31.96 (5.37)	32.65 (4.13)	33.38 (4.91)	0.088	31.88 (5.23)	32.60 (4.31)	33.50 (4.88)	**0.043**
**WC (cm)**		105.51 (9.15)	106.34 (9.68)	108.25 (10.02)	0.092	105.34 (10.51)	106.82 (8.73)	107.95 (9.59)	0.127	105.26 (10.05)	106.21 (8.44)	108.63 (10.19)	**0.026**
**Height (cm)**		115.08 (9.37)	114.82 (8.63)	114.82 (9.87)	0.975	115.19 (9.32)	113.31 (9.04)	116.13 (9.30)	0.095	114.58 (9.52)	113.97 (8.37)	116.15 (9.82)	0.231
**WHR**		0.92 (0.09)	0.93 (0.07)	0.95 (0.07)	**0.043**	0.91 (0.08)	0.95 (0.08)	0.93 (0.07)	**0.011**	0.92 (0.08)	0.93 (0.08)	0.94 (0.06)	0.296
**FM (kg)**		34.51 (7.57)	33.88 (10.57)	33.12 (8.95)	0.699	35.74 (10.57)	33.31 (8.54)	33.12 (8.68)	0.268	34.24 (8.01)	34.43 (9.80)	33.04 (9.29)	0.626
**FFM (kg)**		58.82 (12.18)	62.86 (12.83)	64.71 (11.49)	**0.026**	60.86 (12.92)	62.57 (12.44)	62.79 (12.06)	0.684	59.16 (12.31)	61.50 (12.75)	64.80 (11.672)	**0.037**
**BMR (kcal)**		7228.90 (1602.91)	7996.58 (1513.40)	8097.51 (1680.69)	**0.049**	7778.58 (1520.56)	7950.70 (1473.04)	7852.78 (1787.09)	0.842	7547.92 (1420.28)	7755.70 (1697.38)	8130.95 (1642.48)	0.122
**PA (min/week)**		1653.96 (2786.17)	2405.45 (3498.22)	2371.40 (3287.67)	0.354	2031.22 (2784.99)	1773.48 (2654.86)	2541.14 (3797.19)	0.352	1694.89 2441.22	1793.54 2441.22	2754.25 2441.22	0.108
**MetS [n(%)] Yes**		49 (43.20)	40 (35.39)	46 (40.70)	0.703*	46 (41.10)	52 (46)	37 (32.70)	0.201*	48 (42.90)	43 (38)	44 (38.90)	0.550*

BMI, Body mass index; WC, Waist Circumference; WHR, waist to hip ratio; FM, Fat Mass; FFM, Fat Free Mass; BMR, Basal Metabolic Rate; PA, Physical Activity; MetS, metabolic syndrome; all data are mean (± SD) except MetS that is presented as number and percent. P values derived from One-Way ANOVA with Tukey’s post-hoc comparisons. P* values derived from chi-squared test

**Table 2:** Energy-adjusted dietary intakes of study participants across different tertiles of dietary choline, betaine and total choline and betaine intake

Dietary component intake	Total choline				Total betaine				Total choline and betaine			
	1st tertile (n = 112)	2nd tertile (n = 113)	3rd tertile (n = 113)	P	1st tertile (n = 112)	2nd tertile (n = 113)	3rd tertile (n = 113)	P	1st tertile (n = 112)	2nd tertile (n = 113)	3rd tertile (n = 113)	P
**Glycero- phospho-choline**	36.10 (13.17)	51.32 (15.26)	79.50 (27.79)	< 0.001	46.02 (19.10)	54.88 (25.33)	66.11 (30.74)	< 0.001	39.28 (14.65)	52.60 (19.80)	75.07 (29.82)	< 0.001
**Phospho-choline**	9.24 (3.99)	12.30 (3.84)	18.82 (6.41)	< 0.001	12.30 (5.05)	12.94 (5.72)	15.13 (7.57)	0.002	10.34 (4.57)	12.67 (4.76)	17.35 (7.12)	< 0.001
**Phosphatidyl-choline**	78.59 (24.23)	125.79 (30.43)	207.36 (70.12)	< 0.001	108.52 (44.46)	139.43 (76.47)	164.06 (74.80)	< 0.001	86.51 (29.483)	125.80 (40.83)	199.51 (76.23)	< 0.001
**Sphingomyelin**	6.75 (2.29)	10.48 (2.93)	17.21 (5.54)	< 0.001	9.55 (3.97)	11.92 (6.41)	12.99 (6.18)	< 0.001	7.72 (2.73)	10.60 (4.13)	16.13 (6.31)	< 0.001
**Protein (g/day)**	70.82 (17.15)	93.77 (18.45)	133.88 (38.35)	< 0.001	81.5825 (24.18)	95.8602 (30.48)	121.6646 (42.68)	< 0.001	72.85 (19.22)	94.10 (21.55)	131.76 (39.04)	< 0.001
**Fat (g/day)**	71.31 (26.92)	93.54 (34.23)	136.36 (51.15)	< 0.001	80.29 (37.19)	96.58 (39.98)	124.98 (52.19)	< 0.001	71.6 (26.38)	97.30 (42.80)	132.55 (47.99)	< 0.001
**Carbohydrate (g/day)**	341.18 )109.99)	428.57 (117.80)	582.15 (176.52)	< 0.001	379.20 (138.95)	410.65 (126.43)	564.70 (179.39)	< 0.001	340.2 (117.48)	430.29 (118.96)	582.10 (170.36)	< 0.001
**Total Fiber (g/day)**	51.87 (27.32)	63.39 (31.45)	99.03 (53.08)	< 0.001	35.55 (9.13)	52.45 (13.62)	108.26 (44.69)	< 0.001	37.51 (10.95)	58.94 (21.12)	104.56 (48.56)	< 0.001
**Saturated fatty acids (mg/day)**	20.20 (7.63)	26.98 (9.21)	40.65 (17.80)	< 0.001	25.04 (12.95)	28.27 (12.35)	34.73 (17.66)	< 0.001	21.36 (8.519)	28.41 (13.29)	38.16 (16.91)	< 0.001
**Iron (mg/day)**	18.37 (11.05)	22.11 (6.32)	30.83 (10.31)	< 0.001	18.1 (5.93)	21.15 (5.59)	32.02 (13.24)	< 0.001	16.86 (5.17)	22.63 (10.58)	31.76 (9.81)	< 0.001
**Magnesium (mg/day)**	392.22 (122.58)	516.88 (133.99)	717.59 (270.72)	< 0.001	457.36 (146.88)	510.42 (171.51)	660.61 (294.92)	< 0.001	403.15 (130.302)	524.86 (147.159)	698.34 (276.97)	< 0.001
**Zinc (mg/day)**	10.36 (2.92)	13.86 (3.26)	20.10 (8.63)	< 0.001	12.18 (3.89)	14.05 (4.92)	18.1 (9.19)	< 0.001	10.83 (3.30)	13.96 (3.80)	19.51 (8.80)	< 0.001
**Phosphorus (mg/day)**	1281.90 (323.25)	1714.54 (377.19)	2407.33 (651.53)	< 0.001	1535.21 (484.41)	1722.86 (532.91)	2151.82 (773.77)	< 0.001	1348.53 (390.59)	1725.58 (423.680)	2328.73 (699.16)	< 0.001
**Calcium (mg/day)**	887.89 (285.26)	1201.82 (356.38)	1774.44 (602.80)	< 0.001	1059.18 (425.06)	1175.92 (437.44)	1633.38 (648.53)	< 0.001	913.55 (311.04)	1210.49 (395.10)	1738.43 (608.04)	< 0.001
**Potassium (mg/day)**	3341.69 (1169.24)	4466.70 (1314.78)	6389.50 (2193.42)	< 0.001	4300.97 (1740.11)	4456.82 (1795.46)	5458.71 (2374.35)	< 0.001	3652.92 (1446.56)	4551.11 (1596.34)	5993.80 (2278.87)	< 0.001
**VitaminB9 (µg/day)**	541.49 (157.50)	665.22 (192.78)	956.52 (323.49)	< 0.001	555.41 (191.91)	645.58 (151.54)	963.46 (325.02)	< 0.001	516.69 (149.97)	659.75 (153.84)	984.76 (307.67)	< 0.001
**VitaminB12 (µg/day)**	3.02 (2.093)	5.46 (7.38)	7.52 (6.22)	< 0.001	4.34 (4.54)	5.22 (5.19)	6.48 (7.64)	0.027	3.43 (2.64)	5.23 (6.05)	7.35 (7.52)	< 0.001
**Vitamin A (RAE/day)**	557.88 (289.11)	895.65 (738.33)	1248.92 (740.10)	< 0.001	799.14 (589.25)	882.21 (631.58)	1026.26 (805.70)	0.043	604.39 (362.23)	914.70 (672.12)	1184.38 (815.53)	< 0.001
**Vitamin D (µg/day)**	1.33 (1.05)	1.85 (1.26)	2.91 (1.69)	< 0.001	2.02 (1.39)	1.96 (1.40)	2.13 (1.72)	0.718	1.60 (1.187)	1.92 (1.39)	2.58 (1.73)	< 0.001
**Vitamin K (µg/day)**	185.8814 (188.38)	224.9417 (167.92)	347.7095 (321.03)	< 0.001	195.10 (151.45)	256.65 (288.57)	307.21 (261.42)	0.003	163.14 (135.42)	241.93 (195.37)	352.61 (324.70)	< 0.001
**Vitamin E (mg/day)**	12.20 (5.99)	16.12 (8.33)	20.38 (8.38)	< 0.001	13.40 (6.065)	15.50 (7.794)	19.84 (9.49)	< 0.001	11.98 (5.37)	16.38 (8.095)	20.33 (8.91)	< 0.001

**Table 3:** Biochemical parameters of study participants across different tertiles of dietary choline, betaine and total choline and betaine intake

Variables	Total choline					Total betaine					Total choline and betaine				
	T1 (n = 112)	T2 (n = 113)	T3 (n = 113)	P*	P**	T1 (n = 112)	T2 (n = 113)	T3 (n = 113)	P*	P**	T1 (n = 112)	T2 (n = 113)	T3 (n = 113)	P*	P**
**SBP (mmHg)**	123.29 (15.33)	122.95 (14.60)	121.84 (18.87)	0.785	**< 0.001**	125.63 (14.99)	122.61 (13.42)	119.86 (19.58)	**0.029**	**< 0.001**	125.35 (14.94)	121.46 (14.21)	121.29 (19.22)	0.109	**< 0.001**
**DBP (mmHg)**	82.70 (10.99)	81.42 (10.94)	80.75 (13.08)	0.451	0.286	83.69 (10.31)	81.11 (10.85)	80.09 (13.50)	**0.049**	0.199	83.57 (10.20)	81.13 (11.64)	80.18 (12.95)	**0.051**	0.248
**TC (mg/dL)**	196.12 (41.08)	193.06 (33.31)	186.22 (35.15)	0.118	0.351	195.64 (40.92)	191.20 (35.37)	188.51 (33.61)	0.341	0.907	195.79 (41.88)	193.18 (33.02)	186.41 (34.49)	0.142	0.184
**TG (mg/dL)**	155.64 (104.58)	151.60 (84.17)	146.54 (90.96)	0.766	0.065	157.53 (85.26)	151.83 (109.28)	144.41 (83.76)	0.573	0.061	156.93 (97.74)	154.35 (105.90)	142.50 (73.75)	0.467	0.059
**HDL-C (mg/dL)**	43.33 (9.70)	44.20 (9.71)	43.06 (9.18)	0.643	0.398	43.91 (10.29)	43.43 (8.89)	43.26 (9.40)	0.869	0.452	43.88 (10.29)	43.40 (9.15)	43.33 (9.15)	0.897	0.466
**LDL-C (mg/dL)**	127.55 (33.84)	124.07 (29.87)	119.25 (31.94)	0.149	0.157	127.90 (34.88)	123.30 (30.43)	119.64 (30.23)	0.152	0.162	128.54 (34.10)	124.81 (30.87)	117.51 (30.19)	**0.031**	0.055
**Glucose (mg/dL)**	90.44 (12.71)	92.26 (14.52)	95.50 (27.36)	0.141	0.104	91.78 (15.67)	94.63 (24.31)	91.85 (17.22)	0.456	0.066	90.73 (15.98)	94.04 (23.84)	93.48 (17.56)	0.396	0.065
**Insulin (µIU/mL)**	15.51 (10.06)	16.30 (10.84)	16.25 (17.87)	0.918	0.256	17.11 (11.37)	16.43 (11.52)	14.85 (16.20)	0.528	0.247	15.87 (10.15)	17.09 (12.39)	15.25 (16.41)	0.664	0.229
**HOMA-IR**	3.56 (2.52)	3.68 (2.32)	3.96 (4.38)	0.721	0.245	3.95 (3.00)	3.93 (3.01)	3.41 (3.55)	0.448	0.238	3.58 (2.49)	4.11 (3.35)	3.54 (3.62)	0.453	0.233
**QUICKI**	0.33 (0.04)	0.33 (0.03)	0.33 (0.04)	0.681	0.335	0.32 (0.03)	0.33 (0.04)	0.33 (0.03)	0.226	0.380	0.33 (0.040	0.33 (0.04)	0.33 (0.03)	0.514	0.302

SBP, systolic blood pressure; DBP, diastolic blood pressure; TC, total cholesterol; TG, triglyceride; HDL-C, high density lipoprotein cholesterol; LDL-C, low density lipoprotein cholesterol; HOMA-IR, homeostatic model of insulin resistance; QUICKI, quantitative insulin sensitivity check index); P* values are obtained from ANCOVA model after adjustment for the confounding effects of age, sex, BMI and physical activity. P** values are obtained from ANCOVA model after adjustment for the confounding effects of age, sex, BMI, physical activity, history of CVD, smoking and total energy intake

**Table 4:** Biochemical parameters of study participants based on MetS status across different tertiles of dietary choline, betaine and total choline and betaine intake

			SBP (mmHg)	DBP (mmHg)	TC (mg/dL)	TG (mg/dL)	HDL-C (mg/dL)	LDL-C (mg/dL)	Glucose (mg/dL)	Insulin (µIU/mL)	HOMA-IR	QUICKI
**MetS**	Total choline	T1	129.35 (17.82)	85.16 (13.82)	202.06 (39.00)	179.87 (92.20)	38.67 (7.90)	129.74 (34.27)	96.45 (17.01)	16.13 (8.21)	3.77 (1.83)	0.32 (0.03)
		T2	128.60 (16.41)	83.26 (12.66)	200.47 (33.01)	201.43 (67.54)	39.47 (6.37)	122.68 (31.38)	99.91 (21.57)	18.83 (9.27)	4.60 (2.24)	0.31 (0.03)
		T3	123.48 (26.34)	79.18 (17.97)	199.25 (40.13)	164.11 (69.38)	41.29 (8.08)	127.22 (37.32)	113.74 (45.75)	21.84 (28.77)	5.95 (6.88)	0.31 (0.03)
		P*	0.392	0.997	0.643	0.146	0.077	0.719	0.096	0.358	0.276	0.488
	Total betaine	T1	133.57 (18.45)	85.96 (11.67)	203.46 (46.68)	194.84 (95.09)	40.61 (10.19)	130.06 (40.15)	101.15 (22.90)	20.10 (12.04)	4.92 (3.25)	0.31(0.02)
		T2	123.15 (13.13)	78.57 (11.23)	197.73 (27.88)	175.73 (60.02)	40.07 (5.90)	122.36 (28.38)	108.30 (44.90)	17.12 (9.36)	4.67 (3.60)	0.32 (0.03)
		T3	125.06 (26.47)	83.27 (19.73)	200.82 (36.48)	172.58 (78.84)	38.75 (6.11)	128.13 (34.07)	100.44 (21.93)	19.14 (26.71)	4.63 (5.74)	0.32 (0.03)
		P*	0.131	0.476	0.509	0.131	0.187	0.506	0.503	0.396	0.300	0.447
	Total choline and betaine	T1	130.82 (19.41)	84.28 (12.53)	200.57 (40.25)	186.39 (92.17)	39.32 (8.56)	126.55 (34.24)	97.82 (23.95)	16.67 (8.73)	3.86 (1.84)	0.32 (0.03)
		T2	126.13 (13.53)	83.86 (15.34)	202.90 (35.59)	174.54 (71.14)	40.86 (7.74)	130.01 (37.35)	110.09 (47.23)	23.21 (11.99)	6.40 (4.29)	0.30 (0.03)
		T3	124.64 (25.46)	80.25 (17.04)	199.19 (37.00)	180.03 (73.14)	39.41 (6.56)	125.00 (32.91)	103.16 (21.47)	17.59 (25.97)	4.34 (5.62)	0.32 (0.03)
		P*	0.393	0.938	0.662	0.139	0.114	0.707	0.109	0.497	0.383	0.518
**None-MetS**	Total choline	T1	117.65 (11.60)	76.56 (8.52)	194.52 (40.03)	122.43 (87.35)	47.36 (8.52)	124.74 (37.09)	88.97 (9.42)	15.08 (11.20)	3.41 (2.90)	0.33 (0.03)
		T2	116.39 (13.35)	77.47 (10.27)	186.90 (34.41)	117.91 (60.02)	47.24 (10.06)	121.58 (28.77)	88.96 (9.33)	15.34 (11.29)	3.32 (2.26)	0.33 (0.03)
		T3	116.27 (16.29)	77.18 (10.58)	177.24 (31.45)	108.15 (50.49)	47.89 (8.74)	112.94 (28.30)	87.77 (12.41)	13.64 (8.43)	3.02(1.96)	0.33 (0.03)
		P*	**0.001**	0.072	0.222	**0.001**	0.084	0.149	0.202	0.051	0.056	**0**.614
	Total betaine	T1	117.00 (12.53)	78.02 (8.63)	189.12 (41.43)	132.75 (74.75)	46.47 (10.57)	122.69 (33.97)	87.04 (8.84)	15.52 (10.79)	3.43 (2.75)	0.33 (0.03)
		T2	115.94 (12.36)	77.00 (10.33)	186.29 (34.08)	100.21 (33.07)	46.03 (8.58)	122.26 (31.53)	88.78 (10.02)	16.07 (12.55)	3.55 (2.61)	0.33 (0.04)
		T3	114.95 (16.15)	76.53 (10.48)	182.47 (32.01)	115.10 (75.12)	46.51 (8.65)	114.74 (28.99)	88.09 (12.03)	12.93 (7.55)	2.86 (1.70)	0.33 (0.03)
		P*	**0.001**	**0.038**	0.129	**0.005**	0.102	0.144	0.115	0.071	0.084	0.538
	Total choline and betaine	T1	117.41 (12.69)	78.47 (8.70)	193.06 (42.53)	123.04 (63.09)	48.86 (10.21)	126.86 (35.55)	88.00 (10.58)	15.37 (10.98)	3.40 (2.81)	0.33 (0.03)
		T2	114.48 (12.46)	76.00 (9.90)	186.93 (32.46)	119.50 (79.71)	47.13 (8.44)	120.33 (29.86)	88.48 (12.34)	14.77 (11.82)	3.24 (2.43)	0.33 (0.03)
		T3	116.01 (16.16)	77.16 (10.69)	178.78 (31.72)	106.67 (51.79)	48.88 (8.92)	112.96 (28.41)	87.27 (12.34)	14.05 (8.17)	3.13 (1.88)	0.33 (0.03)
		P*	**< 0.001**	**0.030**	0.148	**0.002**	0.092	0.130	0.420	**0.046**	0.057	0.673

MetS, metabolic syndrome; SBP, systolic blood pressure; DBP, diastolic blood pressure; TC, total cholesterol; TG, triglyceride; HDL-C, high density lipoprotein cholesterol; LDL-C, low density lipoprotein cholesterol; HOMA-IR, homeostatic model of insulin resistance; QUICKI, quantitative insulin sensitivity check index); P-values are obtained from ANCOVA model after adjustment for the confounding effects of age, sex, BMI, physical activity, history of CVD, smoking and total energy intake

**Fig. 1:**
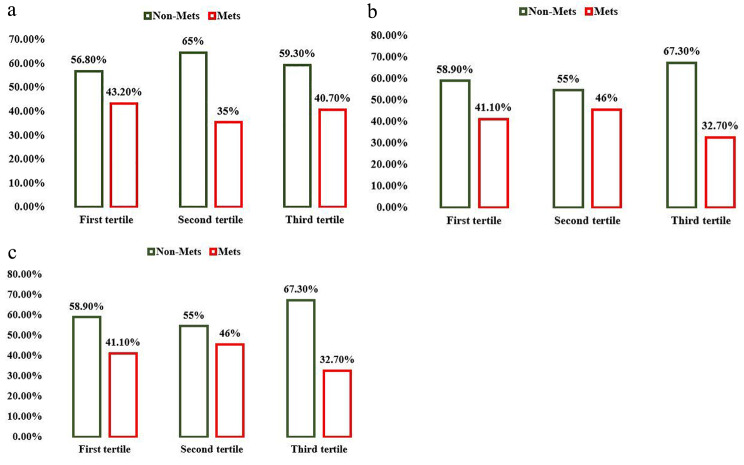
The prevalence of metabolic syndrome in different choline, betaine and total choline and betaine intake categories (P = 0.703, 0.201 and 0.550 respectively, by chi-square analysis)

There was a reduction in the prevalence of MetS by increase in tertiles of dietary choline, betaine and total choline and betaine intakes among participants (Fig. 1).

## Discussion

The results of the current study showed that higher dietary choline and betaine intakes was associated with increased BMI and WHR among obese individuals, although FFM and BMR were also greater in higher tertiles of dietary choline and betaine intakes. Moreover, reduced blood pressure and LDL concentrations and a non-significant reduction in TC and TG levels were also observed even after adjustment for the confounding effects of age, BMI, physical activity level, smoking, history of CVD and total energy intake.

Similar to our findings, increased BMI and WHR by increased dietary choline intake were also observed in the study by Golzarand M et al., [36] and Dibaba D et al., [37] in general population. While in several other studies no significant difference or reduced BMI level was reported in different dietary betaine or choline categories [34, 35]. It seems that the inconsistency in results of different studies is due to difference in the general and demographic characteristics of the studies’ populations. We enrolled obese individuals and observed a difference in BMI between tertiles of dietary choline and total choline and betaine intakes after adjustment for dietary energy intake. In the study by Wu G et al. [53], feeding rats with choline-deficient diet led to body weight gain and reduced fat mass among eight-week-old male ob/ob mice; the observed weight gain was due to increased adipose tissue lipolytic activity and enhanced expression of active hormone-sensitive lipase by choline-deficient diet. In another study by Raubenheimer PJ et al., [54, 55] total weight gain after feeding choline-deficient diets in rats was lower than choline-supplemented diets. Although BMI increased, but it seems that body composition rather than BMI is a better reflection of anthropometric changes in our adult population, because increased dietary choline and betaine intakes was associated with increased FFM and BMR and a non-significant reduction in fat mas; this finding was very interesting and confirming the previous study by Gao X et al., reporting higher dietary choline and betaine intakes was associated with better body composition among the adult Canadian population [34]. Reduced blood pressure due to increased dietary betaine and total choline and betaine intakes in our study was similar to previous studies; in one population- based cross-sectional study among individuals aged more than 20 years old, dietary choline intake was inversely associated with incidence of hypertension among women [*n* = 4748; odds ratio (OR): 0.89; 95% CI: 0.77, 1.02] [56]. In another study by Taesuwan S et al., [57], dietary choline intake was inversely associated with blood pressure in a cross-sectional study of National Health and Nutrition Examination Survey (NHANES). The proposed mechanisms for protective role of dietary choline and betaine against hypertension is endogenous production of a phosphatidylcholine (PC) molecule that exerts anti-hypertensive effects due to its high docosahexaenoic acid (DHA) content; it is shown that PC also reduces heart rate and improves vascular reactivity in human [57, 58]. Also, choline improves vagal activity and inhibits the inflammatory response in spontaneous hypertension and therefore, reduces the consequent cardiovascular damage in hypertension [59–61].

In our study, increased dietary choline and betaine intakes were also associated with reduced TC, TG and LDL concentrations. Although, reduced TG and TC were not statistically significant, but the reduction was clinically meaningful. Choline supplementation normalizes cholesterol metabolism and the expression of genes involved in cholesterol transport and esterification [62]. Similar to our study, in the study by Roe J et al. serum betaine but not choline was associated with favorable cardio-metabolic risk factors (e.g. lower LDL and TG) among older adults [63]. In another study choline supplementation reduced serum cholesterol and LDL concentrations in patients with type 2 diabetes mellitus (T_2_DM) [64]. While several other studies found a positive association between dietary choline intake or choline supplementation and serum lipids; in the study by Pary AV et al., [65], a weak positive association between dietary choline intake and serum LDL was reported only up to an intake of ± 250 mg/day. In an experimental model, choline deficiency reduced all kinds of serum lipids among female rats [66]. In one study, three eggs intake per day for four weeks, as the main dietary choline source, increased total cholesterol, HDL, and LDL cholesterol in healthy volunteers [67], while in another study, phosphatidylcholine supplementation in healthy humans did not alter serum cholesterol but increased TG levels [68]. These findings indicate that choline form (e.g. its biochemical structure, and dietary or supplemented choline) and dosage are important determinants of its health effects.

Concerning the limitations of the current study, the study’s cross-sectional design makes it challenging to draw conclusions about causality; longitudinal investigations are required to clarify the cause-effect relationships between dietary choline and betaine intake, and cardio-metabolic risk factors. Also, we used semi-quantitative FFQ for dietary assessment that because of its subjective nature, it might stem for recall bias; however, the FFQ’s validity and reliability was confirmed in the previous studies. The multiple variables investigated as well as the relatively high number of samples are other strengths of this study.

**Fig. 2:**
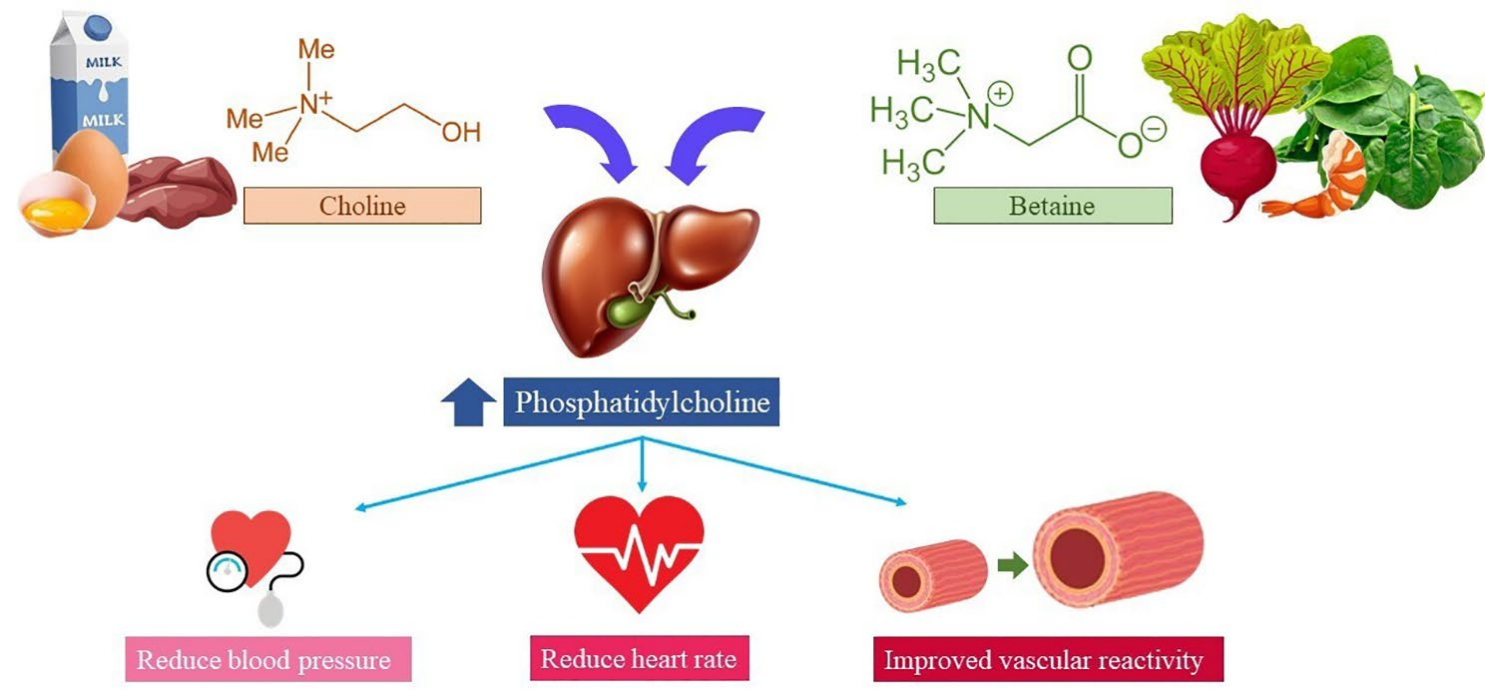
Summarized beneficial effects of choline and betaine on blood pressure observed in the current study

In conclusion, dietary choline and betaine intakes in obese individuals were associated with lower levels of blood pressure and low density lipoprotein (LDL) concentrations. The summarized beneficial effects of choline and betaine is presented as graphical abstract in Fig. 2. Due to great between-study heterogeneity about the health effects of dietary choline and betaine in different populations, further studies are warranted to expand these findings to different geographical distributions.

## Data Availability

The datasets used and/or analyzed during the current study available from the corresponding author on reasonable request.
